# Functional alterations of astrocytes in mental disorders: pharmacological significance as a drug target

**DOI:** 10.3389/fncel.2015.00261

**Published:** 2015-07-06

**Authors:** Yutaka Koyama

**Affiliations:** Laboratory of Pharmacology, Faculty of Pharmacy, Osaka Ohtani UniversityTondabayashi, Osaka, Japan

**Keywords:** astrocyte, schizophrenia, mood disease, drug dependence, neurodevelopmental disorder

## Abstract

Astrocytes play an essential role in supporting brain functions in physiological and pathological states. Modulation of their pathophysiological responses have beneficial actions on nerve tissue injured by brain insults and neurodegenerative diseases, therefore astrocytes are recognized as promising targets for neuroprotective drugs. Recent investigations have identified several astrocytic mechanisms for modulating synaptic transmission and neural plasticity. These include altered expression of transporters for neurotransmitters, release of gliotransmitters and neurotrophic factors, and intercellular communication through gap junctions. Investigation of patients with mental disorders shows morphological and functional alterations in astrocytes. According to these observations, manipulation of astrocytic function by gene mutation and pharmacological tools reproduce mental disorder-like behavior in experimental animals. Some drugs clinically used for mental disorders affect astrocyte function. As experimental evidence shows their role in the pathogenesis of mental disorders, astrocytes have gained much attention as drug targets for mental disorders. In this paper, I review functional alterations of astrocytes in several mental disorders including schizophrenia, mood disorder, drug dependence, and neurodevelopmental disorders. The pharmacological significance of astrocytes in mental disorders is also discussed.

## Introduction

Astrocytes are the most numerous glial cell in the brain and play an essential role in maintaining efficient neurotransmission through the supply of energy metabolites, turnover of neurotransmitters, and establishment of the blood–brain barrier. In earlier studies, astrocytes were not thought to be actively involved in synaptic transmission, but this perception was revised when astrocytes were shown to express receptors for most neurotransmitters, and by which, astrocytic actions are regulated in response to receptor activation. Recent studies have confirmed that astrocytes are more actively involved in synaptic transmission than previously predicted (Perea et al., 2009). Astrocytic mechanisms that regulate synaptic transmission include release of astrocyte-derived neuroactive substances and dynamic regulation of neurotransmitter turnover in response to nerve excitation. Moreover, accumulating evidence has revealed specialized actions of astrocytes in the injured brain. One well-studied feature of astrocytes in neuropathological conditions (including acute brain insults and neurodegenerative diseases) is their phenotypic conversion to reactive astrocytes. Following phenotypic conversion, astrocytes function is altered to affect viability and repair of damaged nerve tissue (Sofroniew, 2009; Buffo et al., 2010; Koyama, 2014). Supported by these findings, modulation of astrocytic pathophysiological function was predicted to have beneficial actions on protection and repair of injured nerve tissue. Subsequent experiments demonstrated the effectiveness of this strategy using neuroprotective drugs (Acarin et al., 2001; Cifra et al., 2011; Tsuda et al., 2011; Carbone et al., 2012), and the pharmacological significance of astrocytes as a drug target for acute brain insults and neurodegenerative diseases is now accepted.

Dysfunction of monoamine- or L-glutamate (L-Glu)-mediated synaptic transmission in particular brain regions is known to be a primary pathogenic cause of many mental disorders (Herberg and Rose, 1990; Lee et al., 2007; Laruelle, 2014; Perez and Lodge, 2014). Prompted by the concept that astrocytes are more actively involved in synaptic transmission, many studies have been carried out to relate astrocyte dysfunction with mental disorders. Nervous tissue dysplasia during embryonic and postnatal brain development has also been suggested to induce mental disorders in adults. Although neuronal degeneration is not a common pathological feature in mental disorder patients, morphological and functional observations reveal alterations in astrocyte density and gene expression in several disorders (Cotter et al., 2002; Stockmeier et al., 2004; Choudary et al., 2005; Madeira et al., 2008; Habl et al., 2009; Beardsley and Hauser, 2014). Moreover, many observations have shown that modulation of astrocyte function using gene manipulation and pharmacological tools affects mental disorder-like behavior in experimental animals (Ballas et al., 2009; Basu et al., 2009; Labrie et al., 2010; Sun et al., 2012; Yang et al., 2012; Kong et al., 2014). Additionally, studies on the therapeutic mechanisms of drugs currently used to treat mental disorders found that their beneficial effects are mediated via astrocytic mechanisms (Table 1). From these findings, astrocytes were suggested to play an important role in the etiology of mental disorders. In the present clinical field, several types of effective drugs are used for care of patients with mental disorders. However, further development of psychiatric drugs will be required. Thus, the use of astrocytic cell pathways was proposed as a novel strategy in mental disorder etiology, as well as in the mechanisms of neuroprotective drugs.

**Table 1:** **Drugs showing therapeutic effects on mental disorders by modulation of astrocytic functions**.

Drugs	Actions on astrocytes	Mental disorder	Reference
ALX5497, NEPS	Inhibition of D-Ser uptake	Schizophrenia	Lipina et al. (2005) Karasawa et al. (2008) Kawaura et al. (2014)
Compound 8, CBIO, AS05728	Inhibition of DAAO	Schizophrenia	Adage et al. (2008) Hashimoto et al. (2009) Smith et al. (2009)
Clozapine	Stimulation of D-Ser release	Schizophrenia	Tanahashi et al. (2012)
Riluzole	Increases in L-Glu transport	Depression	Banasr et al. (2010)
Desipramine, Fluoxtine, Mianserin, Chromipramine, Paroxetine	Productions of neurotrophic factors	Depression	Hisaoka et al. (2001) Mallei et al. (2002) Bachis et al. (2008) Allaman et al. (2011) Liu et al. (2012) Kittel-Schneider et al. (2012)
Amitriptyrine, Fluoxetine, Duloxetine	Increases in CX43 expression	Depression	Sun et al. (2012) Morioka et al. (2014)
MS-153	Increases in L-Glu transport	Drug dependence	Nakagawa et al. (2005)
Cabergoline	Production of GDNF	Drug dependence	Ohta et al. (2003, 2004)
Fenobam, AFQ056	mGluR5 antagonism	Fragile X syndrome	Levenga et al. (2011) Vinueza Veloz et al. (2012)

This paper reviews recent studies on the possible roles of astrocytes in the pathogenesis of mental disorders i.e., schizophrenia, mood disorders, drug dependence, and mental retardation (Rett syndrome and fragile X syndrome, FXS). The pharmacological significance of astrocytes as drug targets for mental disorders is also discussed.

## Novel Concepts of Astrocyte Function

Astrocytes are known to play a supporting role in synaptic transmission including maintenance of the ionic balance in extracellular fluid, supply of energy metabolites to neurons, and reducing transmitters released into the synaptic cleft (Parpura et al., 2012). To undertake these supporting roles, astrocytes have many specific transporters and neurotransmitter metabolizing enzymes. During regulation of synaptic transmission by L-Glu, astrocytes take up synaptic L-Glu through highly expressed excitatory amino acid transporters (EAAT-1 and EAAT-2). Subsequently, L-Glu is metabolized to L-glutamine by an astrocyte-specific enzyme, glutamine synthetase (GS). Release of astrocytic L-glutamine is used as a neuronal L-Glu precursor, and this interplay between neurons and astrocytes is known as the glutamine cycle. Specific transporters and metabolizing enzymes for other neurotransmitters are also expressed in astrocytes. Expression levels of astrocytic transporters and metabolizing enzymes are not static, but are dynamically regulated in response to synaptic activity. This enables astrocytes to effectively support synaptic transmission. Aside from their supporting role, the concept that astrocytes are more actively involved in synaptic transmission is being recognized. This concept involves the “tripartite synapse”, with astrocytes surrounding the synaptic cleft as an essential component of the synapse, as well as pre- and post-synaptic neurons, and with part of the pre-synaptic signal circumvented via astrocytes to modulate the direct signal to the post-synaptic neuron (Perea et al., 2009). Evidence to support this includes the discovery of “gliotransmitters”. The term “gliotransmitter” is used to describe neuroactive substances released from astrocytes in response to a pre-synaptic signal. Astrocytes excited by L-Glu and adenosine triphosphate (ATP), release L-Glu, ATP, adenosine, D-serine (D-Ser), and eicosanoids in a Ca^2+^ dependent mechanism. Because of this excitation-induced release and modulatory action on synaptic transmission, these substances are thought to be putative gliotransmitters (Araque et al., 2014). However, regulation of synaptic transmission by gliotransmitters is still controversial in physiological states. While release of gliotransmitters is stimulated in a Ca^2+^-dependent manner, experimental manipulation of increased astrocytic Ca^2+^ failed to affect excitatory synaptic activity in the hippocampus (Fiacco et al., 2007; Petravicz et al., 2008). Moreover, increased astrocytic Ca^2+^ levels in response to pre-synaptic activations were obtained after excitation of post-synaptic neurons (Agulhon et al., 2012). From these findings, Agulhon et al. (2012) proposed that the gliotransmitter role is less significant in physiological states.

In pathological states, astrocyte function is remarkably altered. Specifically, astrocytes are converted to a reactive phenotype in response to brain injury, which is characterized by cell body hypertrophy and increased expression of glial fibrillary acidic protein (GFAP), an astrocyte-specific intermediate filament protein (Sofroniew, 2009; Koyama, 2014). Phenotypic conversion to reactive astrocytes is accompanied by altered expression of various functional molecules, such as transporters and neurotransmitter metabolizing enzymes (Buffo et al., 2010). Altered activities of these astrocytic molecules may result in disturbed synaptic transmission and aggravate excitoxicity-induced nerve injury. Several types of soluble factors (e.g., cytokines, chemokines, and neurotrophic factors) that regulate pathophysiological responses in nerve tissue are produced by reactive astrocytes (Hamby and Sofroniew, 2010; Colangelo et al., 2014). Excess production of cytokines and chemokines causes microglial activation, infiltration of blood cells, neural apoptosis, and breakdown of the blood–brain barrier, which exacerbates neuroinflammation in the injured brain. However, reactive astrocytes also produce neurotrophic factors, including brain-derived neurotrophic factor (BDNF) and glial cell line-derived neurotrophic factor (GDNF; Koyama et al., 2003). These astrocyte-derived neurotrophic factors prevent neuronal damage and stimulate neurogenesis, both of which improve dysfunction of the injured brain. By releasing these soluble factors, reactive astrocytes play prominent roles in regulating pathophysiological responses in injured nerve tissue, and suggest that modulation of astrocyte function may be a promising target for neuroprotective drugs, which can treat acute brain insults and neurodegenerative diseases. The neuroprotective action of some drugs in modulating astrocyte function have been observed in animal models of brain ischemia, Parkinson’s disease, and amyotrophic lateral sclerosis (ALS; Acarin et al., 2001; Cifra et al., 2011; Tsuda et al., 2011; Carbone et al., 2012). There have been some excellent review papers on the pharmacological significance of astrocytes as a target for neuroprotective drugs (Darlington, 2005; Hamby and Sofroniew, 2010; Colangelo et al., 2014).

In addition to the release of gliotransmitters and neurotrophic factors, studies have shown novel roles for astrocytic connexin-43 (CX43) and aquaporin-4 (AQP4) in regulating nerve function in both the pathological and physiological state. CX43 is a main component of the astrocytic gap junction (Koulakoff et al., 2008). Intracellular communication through gap junctions enables sharing of cytosolic messengers and excitability between adjacent cells (Scemes and Spray, 2012). In astrocytes, CX43 expression is altered by brain injury (Rouach et al., 2002), which affects the neuroprotective actions and proliferation of astrocytes (Tabernero et al., 2006; Gangoso et al., 2012; Theodoric et al., 2012). Therefore, modulation of CX43-mediated gap junction communication may be a target for neuroprotective drugs. Besides these pathophysiological roles, gap junction activity stimulates the release of various gliotransmitters. Stehberg et al. (2012) found that administration of CX43 inhibitors to the rat basolateral amygdala prevents fear memory consolidation, suggesting CX43 involvement in physiological nerve function. Reduced CX43 expression was observed in patients with major depressive disorder (MDD) and alcohol dependence (Bernard et al., 2011; Miguel-Hidalgo et al., 2014). From these findings, CX43 was proposed to be a target of drugs for mental disorders (Sun et al., 2012; Morioka et al., 2014). AQP4 is a water channel highly expressed in astrocytes. With regards the functional role of astrocytic AQP4, its relationship to brain edema etiology has been investigated (Manley et al., 2000). AQP4 is thought to be involved in glial scar formation at injured nerve tissue and in brain edema, because AQP4 inhibitors stimulate migration of reactive astrocytes (Saadoun et al., 2005; Verkman et al., 2006). Besides these pathological roles, recent studies have suggested novel roles for astrocytic AQP4 in synaptic plasticity and mental disorder pathogenesis. Skucas et al. (2011) found that induction of long term-potentiation was attenuated in the hippocampus of AQP4 null mice. In addition, deletion of astrocytic AQP4 decreased morphine dependence and the anti-depressant actions of fluoxetine (Kong et al., 2009; Yan et al., 2013).

**Figure 1:**
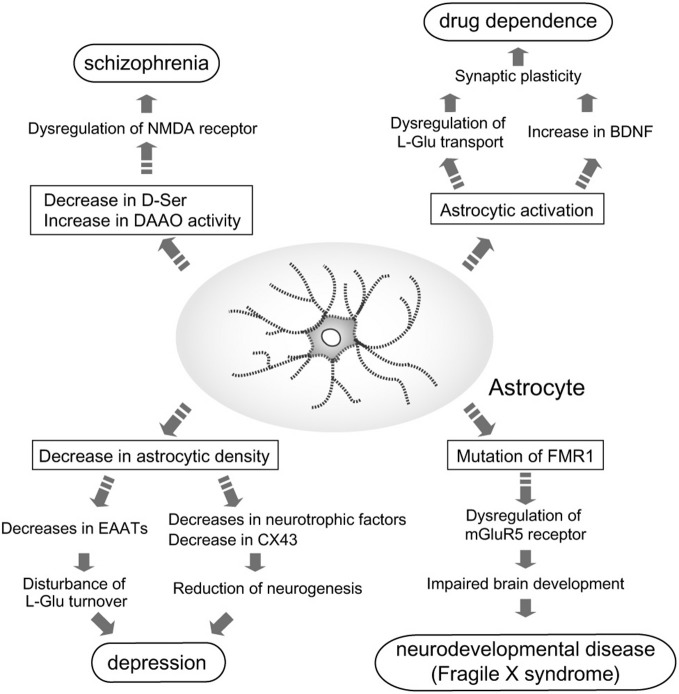
**Astrocyte roles in the pathogenesis of mental disorders**. Pathological roles for astrocytes in induction and/or aggravation of schizophrenia, depression, drug dependence, and fragile X syndrome (FXS) are proposed. In patients with schizophrenia, D-Ser content in the brain is decreased, while D-amino acid oxidase (DAAO) expression is increased. These alterations in D-Ser may cause schizophrenia via dysfunction of *N*-methyl-D-aspartate (NMDA) receptor-mediated signaling. Decreased astrocyte cell number is found in patients with depression. Reduction of astrocytes leads to leads to decreases in neurotrophic factors, CX43 and excitatory amino acid transporters (EAATs). Decreases in these astrocytic molecules cause disturbance of L-Glu turnover and neurogenesis, which may aggravate depression. Many dependence-producing drugs activate astrocytes. Production of brain-derived neurotrophic factor (BDNF) by reactive astrocytes enhances the rewarding effects of psychostimulants. *fragile X mental retardation 1 (FMR1)* is the gene responsible for FXS. Mutations in *FMR1* cause dysfunction of mGluR5 signaling in neurons and astrocytes, which impairs normal brain development.

Because of the identification of these astrocytic functions, the relationship between astrocytes and higher brain functions, including regulation of emotion and mentality, has gained greater attention. Many studies have since been performed to determine the involvement of astrocyte dysfunction in mental disorders (Figure 1), and have shown that astrocytes contribute to the pathogenesis of some disorders.

## Astrocytes in Mental Disorders

### Schizophrenia

Schizophrenia is a mental disease that affects approximately 1% of the population. Its symptoms are hallucination, delusions, thought disorder, flat affect, social withdrawal, and cognitive disorder. Genetic and environmental factors are involved in schizophrenia, although its detailed mechanisms are not fully understood. Drugs with antagonistic potency against dopamine D_2_ receptors are widely used for treating schizophrenia. These antagonists effectively manage the abnormal behavior, and thus dysfunction of midbrain dopamine transmission is generally accepted to underlie the symptoms of schizophrenia. Further studies have shown involvement of L-Glu-mediated excitatory transmission in schizophrenia pathogenesis (Coyle, 2006; Laruelle, 2014). In experimental animals, *N*-methyl-D-aspartate (NMDA) receptor antagonists cause schizophrenia-like behavioral abnormalities, accompanied by dopamine system hyperactivation (Lipina et al., 2005; Karasawa et al., 2008; Bado et al., 2011; Kawaura et al., 2014). Moreover, administration of NMDA antagonists to schizophrenic patients aggravates their symptoms (Javitt and Zukin, 1991; Krystal et al., 1994), suggesting that inhibition of NMDA receptor-mediated transmission facilitates induction of schizophrenia. NMDA receptors have an allosteric site that regulates L-Glu-mediated receptor activation. D-serine is a necessary co-factor for NMDA receptor/channel gating, and enhances the excitatory signal (Balu et al., 2012; Van Horn et al., 2013). The D-Ser biosynthetic enzyme, serine racemase (SR), and D-Ser degradation enzyme, D-amino acid oxidase (DAAO), are both present in brain regions with high NMDA receptor expression (Van Horn et al., 2013). Immunohistochemical observations show that SR locates to astrocytes (Wolosker et al., 1999; Panatier et al., 2006), while D-Ser release from astrocytes is stimulated by excitatory amino acids (Martineau et al., 2014), indicating that D-Ser serves as a gliotransmitter. In schizophrenia patients, D-Ser levels are decreased in cerebrospinal fluid (Hashimoto et al., 2003; Bendikov et al., 2007), whereas DAAO protein and its activity are increased in the hippocampus and cerebrum (Madeira et al., 2008; Habl et al., 2009). Human genetic analysis shows that several polymorphic variants of SR and DAAO are related to increased risk of schizophrenia (Labrie et al., 2009; Caldinelli et al., 2013). Concurrent with these observations, manipulation of brain D-Ser levels induces schizophrenia-like behavior in experimental animals. Basu et al. (2009) reported that genetic deletion of SR causes hyperactivity and impaired memory in mice, accompanied by altered NMDA responses. Further observations of the SR null mouse found morphological and neurochemical abnormalities in the brain, similar to those in schizophrenia (Puhl et al., 2014). In contrast, DAAO deletion reverses schizophrenia-like abnormal behavior in mice with impaired NMDA receptor function (Labrie et al., 2010). The effect of D-Ser and related drugs has been examined in animal models of schizophrenia. Administration of D-Ser, D-Ser reuptake inhibitors (ALX5407 and (R)-(N-[3-(4′-fluorophenyl)-3-(4′-phenylphenoxy)propyl] sarcosine (NFPS)) and DAAO inhibitors ([4H-thieno [3, 2-b]pyrrole-5-carboxylic acid] (compound 8), 5-chloro-benzo[d]isoxazol-3-ol (CBIO) and AS057278) improve impaired pre-pulse inhibition and cognitive defects induced by NMDA antagonists (Lipina et al., 2005; Adage et al., 2008; Karasawa et al., 2008; Hashimoto et al., 2009; Smith et al., 2009; Bado et al., 2011; Kawaura et al., 2014). Therapeutic effects of D-Ser and glycine on negative symptoms of schizophrenia patients have been reported, and more effective drugs for enhancing NMDA receptor signaling should be explored (Tuominen et al., 2005; Tsai and Lin, 2010). Currently, atypical antipsychotics, which improve both positive and negative symptoms, are used for the treatment of schizophrenia. Some atypical antipsychotics (clozapine, olanzapine, and risperidone), but not haloperidol, enhance L-Glu transmission in the prefrontal cortex via NMDA receptors (Ninan et al., 2003; Kargieman et al., 2007). Recently, Tanahashi et al. (2012) showed that clozapine, but not haloperidol, stimulates D-Ser release from astrocytes, suggesting a novel mechanism of atypical antipsychotics in schizophrenia treatment.

### Mood Disorders (Major Depressive Disorder)

Among the mood disorders, morphological and functional alterations of astrocytes are apparent in patients with MDD (Sanacora and Banasr, 2013). Postmortem brain examination of MDD patients shows decreased astrocyte cell number and GFAP protein in the hippocampus (Stockmeier et al., 2004), frontal cortex (Ongür et al., 1998; Cotter et al., 2002), and amygdala (Hamidi et al., 2004). Decreased astrocyte cell number and GFAP protein are reproduced in animal models subjected to chronic unpredictable stress (Heine et al., 2004; Czéh et al., 2006). Administration of L-α-aminoadipate (an astrogliotoxin used as a tool to induce specific astrocytic degeneration) into the rat prefrontal cortex causes depressive-like behavior (Banasr and Duman, 2008). Based on these findings, possible involvement of impaired astrocyte function in the pathogenesis of depression has been investigated. While therapeutic mechanisms of clinically used antidepressants can be explained by the “monoamine hypothesis”, L-Glu transmission has also been considered as a therapeutic target for depression. In rat social interaction and sucrose intake tests, administration of L-Glu transport inhibitors leads to depressive-like behavior (Lee et al., 2007; John et al., 2012), suggesting that impaired L-Glu turnover between astrocytes and neurons causes depression. As well as GFAP, expressions of astrocyte-specific molecules (e.g., EAAT-1, EAAT-2, and GS) are decreased in MDD, along with the reduction in astrocyte cell number (Choudary et al., 2005). As EAAT-1 and EAAT-2 are the main uptake pathways for extracellular L-Glu into astrocytes, decreased EAAT-1 and EAAT-2 expression may cause impaired L-Glu turnover and result in depression. Involvement of impaired L-Glu turnover in depression pathogenesis is supported by the beneficial effect of riluzole in animal models of depression. Riluzole, which is clinically used for ALS treatment, activates L-Glu transporters (Fumagalli et al., 2008). Furthermore, Banasr et al. (2010) found that riluzole reverses decreased GFAP expression in the rat prefrontal cortex and improves depressive-like behavior after chronic unpredicted stress. Although the mechanisms underlying morphological and functional alterations of astrocytes remain to be clarified, the beneficial action of riluzole suggests that modulating L-Glu turnover in astrocytes is a novel strategy for treatment of depression.

Neuronal and glial cell genesis is not limited to the developing brain and can occur in restricted areas of the adult brain, mainly the hippocampus and sub-ventricular zone (SVZ). Many studies have attempted to show correlation between the pathology of neurological disorders and deregulation of cellular genesis in the adult brain. Reduced hippocampal neurogenesis is implicated in the pathogenesis of depression, and as a possible target of antidepressants (Santarelli et al., 2003; Banasr and Duman, 2007). Moreover, recent animal model studies implicate astrogliogenesis in depression pathogenesis. Olfactory bulb dissection can induce depressive-like behavioral changes in rats. Keilhoff et al. (2006) showed that olfactory bulb dissection decreases neural precursor proliferation in the hippocampus and SVZ, which can be rescued by the antidepressant, imipramine. Similarly, chronic social stress decreases astrocyte number and cell volume in the rat hippocampus, which can be reversed by fluoxetine (Czéh et al., 2006). In contrast, electroconvulsive seizures, an effective treatment for severe depression, stimulates astrocyte proliferation in the rat hippocampus and prefrontal cortex (Ongür et al., 2007; Jansson et al., 2009). These findings support the involvement of astrogliogenesis in the pathogenesis of depression. Recently, Kong et al. (2014) found that deletion of AQP4, a water channel protein expressed in astrocytes, aggravates depressive-like behavior and is accompanied by a further reduction in astrocyte cell number and hippocampal neurogenesis. This suggests that astrocytic AQP4 may be a novel target for antidepressants.

Increased production of neurotrophic factors is predicted to be an effective treatment strategy for mood disorders, because they promote neurogenesis, gliogenesis, and synaptic structure remodeling. Levels of BDNF (Dwivedi et al., 2003), GDNF (Otsuki et al., 2008; Zhang et al., 2008), and basic fibroblast growth factor (bFGF; Evans et al., 2004) are decreased in patients with depression, and relates to the reduced hippocampal neurogenesis. Astrocytes are a main source of these neurotrophic factors in pathological brain conditions (Koyama et al., 2003). Administration of antidepressants (e.g., desipramine, fluoxetine, mianserin, clomipramine, and paroxetine) increases production of BDNF, GDNF, and bFGF in the rat hippocampus (Mallei et al., 2002; Martínez-Turrillas et al., 2005; Bachis et al., 2008; Liu et al., 2012), while *in vitro* studies using cultured astrocytes treated with antidepressants shows production of these neurotrophic factors (Hisaoka et al., 2001; Allaman et al., 2011; Kittel-Schneider et al., 2012). Thus, up-regulation of astrocytic trophic factor production may partially underlie the therapeutic actions of presently used antidepressants.

A relationship between CX43, a main component of astrocytic gap junctions, and MDD has been suggested. Reduced brain CX43 expression is observed in MDD patients (Bernard et al., 2011; Miguel-Hidalgo et al., 2014). Inhibition of CX43-mediated gap junction communication causes depressive-like behavior in rodents (Sun et al., 2012). Besides neurotrophic factor production, increased CX43 expression is proposed as a novel mechanism for clinically used antidepressants. Sun et al. (2012) found that fluoxetine and duloxetine increase CX43 expression in rat brain. Moreover, amitriptyline increases CX43 expression by a monoamine-independent mechanism in cultured astrocytes (Morioka et al., 2014).

### Drug Dependence

Repeated abuse of opiates, hypnotics, and psychostimulants leads to drug dependence. It is known that drug-induced alterations in synaptic strength in the mesocorticolimbic dopamine system and modulatory glutamatergic neuronal circuits, both part of the brain reward system, underlie drug dependence (van Huijstee and Mansvelder, 2015). Dependence-producing drugs commonly activate the main pathway of the brain reward system, with dopamine released from neurons in the ventral tegmental area (VTA) to the nucleus accumbens (NAcc) and prefrontal cortex. Studies on the mechanisms underlying drug dependence show a possible role for astrocytes in modulating neurotransmission in the brain reward system (Beardsley and Hauser, 2014). Administration of amphetamine, methamphetamine, cocaine, and morphine induces astrocyte activation and increases GFAP expression in rodent brain (Hebert and O’Callaghan, 2000; Fattore et al., 2002; Pubill et al., 2003; Alonso et al., 2007). Although these astrocytic alterations are not necessarily a common pathological feature shared by other drugs, these observations facilitate examination of the mechanisms underlying drug dependence in the context of astrocyte function.

The L-Glu-mediated neural circuit from the prefrontal cortex to NAcc plays an important regulatory role in the brain reward system (van Huijstee and Mansvelder, 2015). Nakagawa et al. (2005) examined the role of astrocytic L-Glu transporters in mice by co-administrating MS-153, a glutamate transport activator, with morphine, cocaine, or methamphetamine. They found that activation of L-Glu transport attenuates conditioned place preference (CPP) to these drugs. Administration of an adenoviral vector carrying the glutamate transporter 1 (GLT1; EAAT-2) gene into the NAcc also attenuated CPP induction by morphine and methamphetamine (Fujio et al., 2005). Together, these findings suggest there is inhibitory regulation from astrocytic L-Glu transporters on the rewarding effect of dependence-producing drugs.

Astrocyte-derived soluble factors have important roles in regulating synaptic strength and plasticity. The effect of astrocyte-derived factors on susceptibility to drug dependence was examined using conditioned medium from cultured astrocytes. Administration of astrocytic conditioned medium into mouse NAcc caused sensitization of rewarding behavior elicited by methamphetamine and morphine (Narita et al., 2005, 2006), suggesting that astrocytes produce soluble factors that enhance drug dependence. As astrocyte-derived factors affect susceptibility of drug-dependence, the modulatory roles of BDNF and GDNF on rewarding effects of psychostimulants were examined (Ghitza et al., 2010). Enhancement of a rewarding effect by BDNF was first shown by Horger et al. (1999), with chronic BDNF administration into rat NAcc increasing CPP to cocaine. Overexpression of exogenous BDNF and its receptor (TrkB) in rat NAcc also increased CPP to cocaine (Bahi et al., 2008), while mouse BDNF null mutants show reduced CPP (Hall et al., 2003). Positive regulatory roles of BDNF were also suggested from the rewarding effects of morphine and amphetamine (Shen et al., 2006; Vargas-Perez et al., 2009). As had been predicted from animal experiments (Pu et al., 2006; Hatami et al., 2007), a recent study showed that serum BDNF levels in heroin-dependent patients are still higher than those of control groups, even after drug withdrawal (Zhang et al., 2014). The results from viral vector-mediated gene transfer experiments in rodents (Vargas-Perez et al., 2014) propose that enhancement of the BDNF signal in the VTA is related to drug withdrawal aversion.

GDNF was originally discovered as a survival and developmental factor for mesencephalon dopaminergic neurons, and modulates nerve excitation in many brain regions, including the VTA and NAcc (Carnicella and Ron, 2009). In contrast to BDNF, GDNF serves as a negative reinforcement modulator of the rewarding effects of psychostimulants. Administration of GDNF into the rat VTA reduced CPP enhancement to cocaine, while an anti-GDNF neutralizing antibody increased it (Messer et al., 2000). Heterozygous GDNF deletion in mice caused higher sensitivity in CPP and seeking behaviors to methamphetamine than those of wild-type mice (Niwa et al., 2007; Yan et al., 2007). Taking these observations into consideration, a therapeutic effect for drugs enhancing GDNF production in patients with psychostimulant dependence can be expected. Cabergoline, a dopamine D_2_ agonist used for the treatment of hyperprolactinemia and parkinsonism, increases GDNF production in cultured astrocytes (Ohta et al., 2003, 2004) and rat VTA (Carnicella et al., 2009). Cabergoline-induced GDNF production in rat VTA reduced reinforcement of seeking and drinking behavior for alcohol (Carnicella et al., 2009).

### Neurodevelopmental Diseases

Dysplasia of nerve tissue during embryonic and postnatal development underlies some neurological diseases with mental retardation and cognitive defects. During development of the embryonic brain, astrocytes support proliferation and migration of neural precursors, neuronal differentiation, and synaptic formation, although neurogenesis generally precedes maturation of astrocytes from glial precursors (Freeman and Rowitch, 2013). Because of the important role of astrocytes in the developing brain, investigations to explain the etiology of neurodevelopmental diseases by astrocyte dysfunction have been performed (Molofsky et al., 2012; Parpura et al., 2012). A number of studies on two inherited developmental diseases with mental retardation, Rett syndrome and FXS, show that mutations in single genes are responsible for astrocyte dysfunction and impaired brain development.

Rett syndrome, an X-linked neurological disease characterized clinically by distinctive hand movements, seizures, delayed brain and head growth, autism, and mental retardation (Weng et al., 2011), is caused by mutations in a transcription factor, methyl-CpG-binding protein 2 (MeCP2; Samaco and Neul, 2011). In studies using *MeCP2* null mutant mice as a model of Rett syndrome (Chen et al., 2001), conditional MeCP2 expression in postnatal neurons partly reversed behavioral abnormalities (Giacometti et al., 2007; Guy et al., 2007), indicating involvement of reduced neural MeCP2 in pathogenesis of the model. In addition, reduced function of astrocytic MeCP2 is also related to Rett syndrome pathogenesis. *In vitro* experiments by Ballas et al. (2009) found that hippocampal neurons cultured with MeCP2 deleted astrocytes or their conditioned medium, failed to show normal dendritic development. Impaired dendrite formation by astrocytic MeCP2 occurs independent of the presence of neural MeCP2, suggesting that dysregulation of astrocytic soluble factors induced by MeCP2 deletion may relate to induction of Rett syndrome-like phenotypes. Maezawa et al. (2009) reported impairments in BDNF, interleukin-1β, and interleukin-6 production in astrocytes from *MeCP2* deleted mutant mice.

FXS is a neurodevelopmental disease characterized by mental retardation, autism, attention deficit, social anxiety, and specific physical features. One of the genes responsible for FXS, fragile X mental retardation 1 (*FMR1*), is on an X-linked chromosome. Mutations in *FMR1*, with GCC expansions repeats in the promoter region, decrease production of fragile X mental retardation 1 protein (FMRP), which serves as a regulator of local protein translation. Reduced FMRP activity in neurons leads to dysregulation of synaptic protein expression and affects dendrite formation (Bassell and Warren, 2008). *FMR1* gene deletion induces abnormal dendrite elongation and increases spine density in the developing cerebral cortex (Comery et al., 1997; Nimchinsky et al., 2001). Besides neuronal reduction, reduced FMRP activity in astrocytes affects their function during brain development (Jacobs and Doering, 2010; Jacobs et al., 2010). The mechanisms by which reduced FMRP in astrocytes induces abnormal dendrite development were investigated. Yang et al. (2012) found that *FMRP* deletion increases neurotrophin-3 production in astrocytes, which suggests that excess neurotrophic actions underlie abnormalities in dendrite development. The metabotropic glutamate receptor 5 (mGluR5) is predicted to be a therapeutic drug target for FXS (Levenga et al., 2011; Vinueza Veloz et al., 2012; Pop et al., 2014; Scharf et al., 2015). Higashimori et al. (2013) proposed that down-regulation of astrocytic mGluR5 and GLT-1 (EAAT-2) by *FMRP* deletion may cause enhanced neuronal excitation and lead to abnormal dendritic development in FXS mouse models.

## A Perspective of Astrocytes as a Drug Target for Mental Disorders

Supported by considerable experimental evidence, the importance of astrocytic functions during acute brain insults and neurodegenerative diseases is established. Because modulation of astrocytic function has several beneficial actions, astrocytes are a promising target of neuroprotective drugs (Darlington, 2005; Hamby and Sofroniew, 2010; Colangelo et al., 2014). Although neuronal degeneration is generally not observed, disturbance of neurotransmission, abnormal brain development, and remodeling of synaptic structure are found in the brains of patients with mental disorders. Furthermore, morphological and functional alterations of astrocytes are observed in patients with certain mental disorders (Cotter et al., 2002; Stockmeier et al., 2004; Choudary et al., 2005; Madeira et al., 2008; Habl et al., 2009; Beardsley and Hauser, 2014). Besides their role in neurogenesis and synaptic formation during brain development, accumulating evidence shows that astrocytes are an essential component of synaptic transmission (Parpura et al., 2012; Araque et al., 2014). In addition, involvement of astrocyte-specific molecules such as CX43 and AQP4 in higher brain functions is reported (Sun et al., 2012; Xiao and Hu, 2014). Prompted by these findings, many studies have attempted to clarify the role of astrocytes in mental disorders. As described in this review, involvement of astrocytic dysfunction in the pathogenesis of mental disorders is becoming increasingly studied (Figure 1). Thus, the pharmacological significance of astrocytes as a novel drug target for schizophrenia, mood disorders, drug dependence, and neurodevelopmental disorders has been proposed (Table 1). However, despite the accumulating evidence, compared with neurons, there are still many astrocyte-related issues that need to be clarified. These include classification of astrocyte sub-types, differences in properties among brain regions, astrogliogenesis in the developing and adult brain, and the associated regulatory factors. Further investigation of these issues may lead to novel drugs for the treatment for mental disorders.

## Conflict of Interest Statement

The author declares that the research was conducted in the absence of any commercial or financial relationships that could be construed as a potential conflict of interest.

## Abbreviations

L-Glu: L-glutamate
D-Ser: D-serine
GS: glutamine synthetase
GFAP: glial fibrillary acidic protein
BDNF: brain-derived neurotrophic factor
GDNF: glial cell line-derived neurotrophic factor
SR: serine racemase
DAAO: D-amino acid oxidase
SVZ: sub-ventricular zone
bFGF: basic fibroblast growth factor
CX43: connexin-43
AQP4: aquaporin-4
MDD: major depressive disorder
NAcc: nucleus accumbens
VTA: ventral tegmental area
CPP: conditioned place preference
FXS: fragile X syndrome
MeCP2: methyl-CpG-binding protein 2
FMRP: fragile X mental retardation 1 protein.
